# Induced resistance and associated defence gene responses in *Pinus patula*

**DOI:** 10.1186/1753-6561-5-S7-P82

**Published:** 2011-09-13

**Authors:** Katrin Fitza, AA Myburg, Emma Steenkamp, Kitt Payn, Sanushka Naidoo

**Affiliations:** 1 Department of Genetics, University of Pretoria, Pretoria 0002, South Africa; 2 Department of Microbiology, Forestry and Agricultural Biotechnology Institute (FABI), University of Pretoria, Pretoria 0002, South Africa; 3 Mondi South Africa, P.O. Box 12, Hilton, 3245, South Africa

## Background

Plants are able to incite a type of broad spectrum resistance against pathogens upon pre-treatment with biological or chemical inducers. Systemic Acquired Resistance (SAR) and Induced Systemic Resistance (ISR) are two types of induced resistance which lead to the accumulation of specific pathogenesis-related (PR) proteins. Non-pathogenic rhizobacteria are inducing agents for ISR and increased levels of ethylene (ET) and jasmonate (JA) are associated with this pathway, whereas SAR is associated with an increase in salicylic acid (SA) levels [1].

*Pinus patula* and *P. radiata* are commercially planted conifer species in South Africa, but are both highly susceptible to the causal agent of pitch canker, *Fusarium circinatum*. Annually, the forestry sector suffers substantial economic losses due to this disease which affects 20-30% of the planting stock. Bonello *et al*. (2001) showed that repeated mechanical inoculation of *P. radiata* with *F. circinatum* activated induced resistance, enhancing the protection of the tree against subsequent pathogen challenge [2].

Detailed knowledge of the molecular mechanisms underlying induced resistance may be useful to develop strategies to control diseases of pine trees. This study aimed to compare the efficiency of ten biological and chemical inducers in inciting resistance against *F. circinatum.* Additionally the molecular basis of this induced resistance was investigated by analyzing the response of selected putative defence response genes.

## Methods

Ten activators of induced resistance (Bion^®^, Messenger^®^, Chitin, MeJA, *Fusarium oxysporum*, *Pseudomonas fluorescens*, SA, Kannar, *Ralstonia solanacearum* and potassium phosphate monobasic) were compared. A set of 80 *P. patula* seedlings were used per treatment. Inducers were applied at four and six months of age and *F. circinatum* spores (1x10^4^) were used to challenge the seedlings a week after the booster application (six months). Disease severity was assessed six weeks after inoculation by comparing the size of the lesions on treated plants to water control plants. Three inducers that curbed symptoms most successfully were selected for further analysis. A set of 116 plants per treatment were screened weekly for eight weeks. Aerial parts of the six month old plants were harvested for RNA isolation at 24 hrs after the second application. For each treatment, three replicates, with 12 plants per replicate, were harvested. Subsequently, RNA was extracted for the reverse transcriptase quantitative PCR (RT-qPCR) analysis, where four putative defence genes were profiled using the Roche LightCycler® 480 instrument.

## Results and discussion

MeJA, Messenger and chitin treatment resulted in the reduction in symptom severity (results not shown). MeJA, Messenger and the deacetylated version of chitin, chitosan were then tested under stringent inoculation conditions with *F. circinatum* to verify the effectiveness of the inducers. The most promising treatment was chitosan at a concentration of 10 mg/ml, which resulted in a significant reduction in lesion length over a period of 6 weeks. Lesion lengths were converted to percentage live stem (calculated as lesion length divided by plant height multiplied by 100) and are displayed in Table 1.

**Table 1:** Disease progression represented as percentage live stem in 6 month old *P. patula* seedlings during an eight week period post inoculation with *F. circinatum. *The significance levels are relative to the relevant control.

Week	2	3	4	5	6	7	8
**Chitosan (1mg/ml)**	89.73	82.31	76.75	71.43	60.81	61.16	36.10
**Chitosan (10mg/ml)**	91.12*	84.89*	80.60*	72.80*	64.28*	60.32	44.18
**Control**	89.23	82.10	74.48	69.04	58.65	55.15	34.53

*p<0.05, Kruskal-Wallis test.

The defence response elicited by chitosan application was investigated. Four putative genes, representing the onset of SAR and ISR were analyzed (Table 2). Phenylalanine ammonia lyase (*PAL*) had a three-fold accumulation in transcript expression in comparison to uninduced plants (Table 2). The expression level of the 1-deoxy-d-xylulose-5-phosphate synthase (*DXS*) gene, which catalyses the methyl erythriol–phosphate pathway, was down-regulated in comparison to the control. This pathway is important for the production of terpenes, which are building blocks for resin [3]. In previous studies, *PAL* and *DXS* were both shown to be responsive to chitosan. Using the *PAL* gene as a diagnostic marker of the phenylpropanoid pathway, the up-regulation of the transcript suggests that chitosan treatment induces the phenylpropanoid pathway which is known to lead to the production of secondary metabolites to elicit resistance.

**Table 2:** Log2 expression levels of putative defence response genes in *P. patula* 24 hrs after booster treatment with 10 mg/ml chitosan. Three biological replicates were used to calculate significance using the *t*-test.

Gene symbol	Gene Name	Log2 Expression	P-value (t-test)
PAL	Phenylalanine ammonia lyase	1.773	0.028
DXS	l-deoxy-d-xylulose-5-phosphate synthase	-1.142	0.041
FMO	Flavin-dependent monoxygenase	0.486	0.173
PR-3	Chitinase	-0.997	0.199

## Conclusion

The potential of priming *P. patula* to defend itself against pathogen attack was explored. We tested the application of ten different inducers to enhance tolerance to *F. circinatum*. The application of chitosan reduced pitch canker symptoms. Reduced lesion length was observed for a period of six weeks, indicating the activation of induced resistance. Further molecular analysis suggests that the treatment may activate the phenylpropanoid pathway, which is involved in the production of secondary metabolites that have antifungal properties [4]. Entire defence response pathways influenced by chitosan application in *P. patula* will be investigated in subsequent expression profiling assays.
